# Initiating clozapine treatment service and characteristics of clozapine-treated patients in a general hospital in Addis Ababa, Ethiopia

**DOI:** 10.4102/sajpsychiatry.v26i0.1418

**Published:** 2020-06-24

**Authors:** Solomon Teferra

**Affiliations:** 1 Department of Psychiatry, School of Medicine, Addis Ababa University, Addis Ababa, Ethiopia

**Keywords:** clozapine, Ethiopia, mental health service, sub-Saharan Africa, treatment-resistant schizophrenia

## Abstract

**Background:**

At least one-third of patients with schizophrenia suffer from treatment-resistant schizophrenia needing treatment with clozapine. This is the first report on the experience of initiating clozapine service in Ethiopia.

**Aim:**

The aim of this study was to report the experience of setting up clozapine service and describe characteristics of patients treated with clozapine.

**Setting:**

This study was conducted in a general hospital in Addis Ababa, Ethiopia.

**Methods:**

Descriptive summary of the clozapine treatment service and review of characteristics of patients treated with clozapine were conducted. Clinical Global Impression (CGI) Scale and Abnormal Involuntary Movement Scale (AIMS) score were used to measure outcome. Quantitative data were analysed using Statistical Package for the Social Sciences (SPSS) Version 24.

**Results:**

It was possible to provide clozapine treatment in a general hospital using the national guideline. During the first year of initiation of the service, a total of 22 patients were treated. The majority were men (20/22, 90.9%) and in the age group of 30–44 years (11/22, 50%). Indications for clozapine were treatment-resistant schizophrenia (15/22, 68.2%) and tardive dyskinesia (7/22, 31.8%). The average dose of clozapine was 350 mg/day. Common side effects included sedation, constipation and excessive salivation. On CGI Scale, mean severity index score dropped from 5.18 at admission to 3.68 during discharge, and average AIMS score changed from 16.8 to 6.5. None of the patients developed agranulocytosis; however, three patients discontinued because of adverse effects.

**Conclusion:**

Establishing clozapine treatment service was possible in a general hospital in Ethiopia where psychiatric service run by psychiatrists was available. Mechanisms should be in place to ensure adherence to the national guideline.

## Introduction

Schizophrenia remains the second most disabling mental disorder after dementia.^1^ According to the recent global burden of diseases study from reports of 195 countries in the world, schizophrenia was responsible for 13.4 (95% uncertainty interval [UI]: 9.9–16.7) million years of life lived with disability.^2^

Studies from Ethiopia showed lifetime prevalence of schizophrenia at 0.4% and 0.47% in the capital Addis Ababa and rural Butjaira, respectively.^3,4^

Amongst hospitalised patients in the only mental specialised hospital in Addis Ababa, Ethiopia, the majority of patients admitted carry the diagnosis of schizophrenia. According to the descriptive report in 2007 by Fekadu et al., 58% of patients admitted to the hospital were diagnosed with schizophrenia.^5^

Course and outcome studies of schizophrenia in Ethiopia also reported significant number of patients suffering from chronic symptoms. For instance, the 5-year clinical course and outcome report showed that 45% of the participants had continuous symptoms, and a 10-year follow-up study reported tardive dyskinesia in 4.2% of patients.^6,7^ Non-adherence to antipsychotics was also a major problem, side effect being one of the perceived reasons for stopping treatment.^8^

First-generation antipsychotics and electroconvulsive therapy had been the main treatments for schizophrenia in Ethiopia. Two second-generation antipsychotics, resperidone and olanzapine, were recently introduced into the public health service. The introduction of these two second-generation antipsychotics resulted in significant shift in prescription pattern, especially for patients who could not tolerate the first-generation antipsychotics or who were not responding. Despite this new development, a number of patients with schizophrenia continued to suffer from persistent symptoms and side effects such as tardive dyskinesia, which were the result of treatment with first-generation antipsychotics.

The introduction of clozapine was an important milestone for mental health service in the country. Clozapine, a tricyclic dibenzodiazepine drug discovered by a Swiss pharmaceutical company Wander AG in 1959, later acquired by Sandoz in 1967, is considered the most effective antipsychotic drug addressing the treatment needs of at least 30% patients diagnosed with treatment-resistant schizophrenia.^9,10^ Meta-analyses have shown that clozapine results in better improvement in treatment-resistant schizophrenia compared with first-generation and other second-generation antipsychotics.^11^ The problem with clozapine is its many side effects, which need special guideline for treatment and close monitoring, the main problematic adverse effect being agranulocytosis that can be potentially fatal.^12^ Other potentially fatal side effects include myocarditis and thromboembolism.^13,14^ It is also associated with increased risk of seizures, which is often dose-dependent; this risk was found to increase from 1% to 4.4% when the dose was increased from 300 mg/day to 600 mg/day, respectively.^15^ The most common type of seizure was reported to be tonic–clinic.^16^

Sialorrhea, sedation and constipation are common and relatively benign side effects that cause nuisance to patients. Sialorrhea gets worse during sleep; it is believed to be caused by disturbance in swallowing rather than excessive production of saliva.^17,18^ Orthostatic hypotension is also common, which requires close monitoring of blood pressure usually in the first 4–6 weeks of treatment.^19^ A study from South Africa involving 26 participants of the Xosa descent reported high rates of metabolic syndrome and type II diabetes, calling for close monitoring of patients treated with clozapine.^20^. An unpublished thesis from the Eastern Cape province in South Africa reported inadequate monitoring of patients treated with clozapine. According to this report, more than 77% non-compliance rate with haematological monitoring was observed, and all 62 hospitalised patients who were included in the study had no metabolic monitoring.^21^

There is a global standard of practice that countries should follow when introducing clozapine treatment. These include a separate national guideline to determine indication and eligibility and follow the emergence of adverse effects. The introduction of clozapine in Ethiopia followed this process, with the development of a national guideline on prescribing clozapine led by Ethiopian Psychiatric Association (EPA) in 2016.^22^ Following the development of this guideline, treatment was introduced in two facilities: one in Amanuel Mental Specialized Hospital and the other in Zewditu Memorial Hospital, a general hospital, where the author of this article is based. The author oversaw the development of the EPA guideline on clozapine prescription as he was president of EPA during the development of the guideline. According to the EPA guideline, treatment resistance has been defined as follows:

[*W*]hen treatment with at least two antipsychotic medications given at adequate doses for about six weeks fails to relieve the symptoms of psychosis. One of the antipsychotics tried has to be atypical antipsychotic. (p. 3)

The guideline restricts prescription privilege of clozapine to psychiatrists only, and it has to be initiated in the inpatient setting. Details of clinical and laboratory follow-up procedures have been clearly described in the guideline, including weekly test of absolute neutrophil count (ANC) for the first 18 weeks, then every 2 weeks for the next year and then every 4 weeks afterwards. The treatment has to be initiated in an inpatient setting where patients would get optimal care and follow-up by a psychiatrist.^22^

The aim of this report is to share experiences on initiating the clozapine service in a general hospital in Ethiopia and describe the profile of treated patients over a period of 1 year after the treatment was introduced.

To the investigator’s knowledge, this issue has not been studied in Ethiopia thus far; furthermore, there is a dearth of evidence on this issue from sub-Saharan Africa region. It is expected to fill a critical gap in experience and serve as baseline information for further studies.

## Materials and methods

### Setting and participants

The clozapine service was introduced in Zewditu Memorial Hospital, a general hospital located at the heart of Addis Ababa, which was named after Empress Zewditu, the cousin and predecessor of Emperor Haile Selassie. The hospital is under the Addis Ababa Health Bureau. It provides healthcare to residents of Addis Ababa in different specialties, including mental health. The inpatient clozapine treatment unit is located on the third floor housing the internal medicine inpatient service in the hospital’s main building. Two beds were dedicated for clozapine service, adjacent to internal medicine wards. This made consultation with internists very convenient. There is also inpatient detoxification service for addiction and adult psychiatry service on an outpatient basis in the hospital. Clozapine-treated patients receive outpatient follow-up in the same hospital. The hospital is affiliated with the Department of Psychiatry at Addis Ababa University, home institution of the author, and serves as training facility for psychiatry residents and clinical psychology students. The author is the head of psychiatry service and training in the hospital.

### Source of referral and indications for clozapine

We received referrals from our hospital’s psychiatric clinics, and other health facilities providing psychiatric services. Indications for initiating clozapine were ‘treatment-resistant schizophrenia’ as defined by the EPA guideline, and the presence of severe tardive dyskinesia.

### Study design

The author conducted descriptive summary of initiation of the clozapine treatment service, and retrospective chart review of patients treated with clozapine from September 2017 to December 2018.

### Data collection instruments

A structured questionnaire was used to collect socio-demographic and other relevant information. The Clinical Global Impression (CGI) Scale was used to rate global impression on the severity of symptoms before initiation of treatment and measure outcome of treatment, and Abnormal Involuntary Movement Scale (AIMS) was used before and after initiation of clozapine.^23,24^

Training was provided for data collectors by the author. Adequate supervision was provided while data collection was going on. Data were retrieved from patient charts using the structured questionnaire.

### Data analysis

Data analysis was performed using SPSS Version 24.^25^ Descriptive statistics like frequencies, means and medians were calculated.

### Ethical consideration

Ethical approval was obtained from the Scientific Committee of Department of Psychiatry at Addis Ababa University. De-identified data were collected from patient charts by psychiatry residents working in the clozapine clinic.

## Results

### Socio-demographic characteristics

A total of 22 patients treated with clozapine were included in the study. The majority of patients were men (20/22, 90.9%), in the age group of 30–44 years (11/22, 50%), single (19/22, 86.4%), unemployed (14/22, 63.6%) and living with their families (18/22, 81.8%), and most patients (15/22, 68.2%) paid out of pocket for the service they received. Details of socio-demographic characteristics are presented in Table 1.

**TABLE 1:** Socio-demographic characteristics of patients treated with clozapine in Zewditu Memorial Hospital, Addis Ababa, Ethiopia, March 2019.

Variables	Characteristics	Number	%
**Sex**	Males	20	90.9
	Females	2	9.1
**Age group**	18–29 years	7	31.8
	30–44 years	11	50.0
	45+ years	4	18.2
**Residence**	Addis Ababa	18	81.8
	Outside Addis Ababa	4	18.2
**Religion**	Orthodox Christian	7	31.8
	Muslim	3	13.6
	Protestant	2	9.1
	Unspecified	10	45.5
**Educational status**	Primary	5	22.7
	Secondary	8	36.4
	College+ educated	6	27.3
	Unspecified	3	13.6
**Marital status**	Married	2	9.1
	Single	19	86.4
	Unspecified	1	4.5
**Employment status**	Employed	3	13.6
	Unemployed	14	63.6
	Unspecified	5	22.7
**Living arrangement**	Lives with family or relatives	18	81.8
	Lives alone	2	9.1
	Lives with spouse or children	2	9.1
**Health service fee**	Paying out of pocket	15	68.2
	Free	5	22.7
	Other	2	9.1

### Clinical characteristics

Reasons for initiating clozapine included treatment-resistant schizophrenia (15/22, 68.2%) and tardive dyskinesia (7/22, 31.8%). The majority of patients (19/22, 86.4%) continued treatment and got discharged with the drug for outpatient follow-up. The average length of stay in hospital was 37.8 days. The average clozapine dose was 350 mg/day (150 mg in the morning and 200 mg at night). Common side effects encountered include sedation, constipation and excessive salivation. Amongst patients who were diagnosed with tardive dyskinesia, the average AIMS score at admission was 16.8, and at discharge it was significantly reduced to 6.5. On CGI Scale, the mean severity index score dropped from 5.18 at admission to 3.68 at discharge. The mean ANC count at admission was 5158.5 and 5234.9 at discharge. Details of clinical characteristics of the patients are presented in Table 2.

**TABLE 2:** Clinical and laboratory characteristics of patients treated with clozapine in Zewditu Memorial Hospital, Addis Ababa, Ethiopia, March 2019.

Variables	Characteristics	Number	%
**Reason for clozapine treatment**	Treatment-resistant schizophrenia	15	68.2
	Tardive dyskinesia	7	31.8
**Length of stay**	Up to 30 days	9	40.9
	31–60 days	10	45.5
	More than 60 days	3	13.6
	Average length of stay = 37.8 days	-	-
**Clozapine treatment Reasons for discontinuation**	Continued	19	86.4
	Discontinued	3	13.6
	Persistent tachycardia with EEG abnormality	1	4.54
	Cough, fever and tachycardia	1	4.54
	Persistent tachycardia, inadequate improvement	1	4.54
**Clozapine dosage (mean, in mg)**	Starting	-	-
	Morning	12.5†	-
	Evening	12.5	-
	At discharge	-	-
	Morning	147 (median 150)	-
	Evening	186 (median 200)	-
**Common side effects seen in clozapine-treated patients**	Sedation	8	36.36
	Constipation	6	27.27
	Excessive salivation	2	9.09
	Fatigue	1	4.54
**Abnormal Involuntary Movement Scale (AIMS) score (*n* = 10)**	AIMS score at admission (mean)	16.8	-
	AIMS score at discharge (mean)	6.5	-
**Clinical Global Impression (CGI) score**	Severity index score at admission (mean)	5.18‡	-
	Severity index score at discharge (mean)	3.68	-
	Global improvement score at discharge (mean)	2.68§	-
**Absolute neutrophil count in no./mm3 (mean)**	At admission	5158.5	-
	Last count before discharge	5234.9	-

EEG, Electroencephalogram; AIMS, Abnormal Involuntary Movement Scale.

† , Only one patient was started with a morning dose of 12.5 mg of clozapine;

‡ , Score falls between ‘markedly ill’ and ‘severely ill’;

§ , Score falls between ‘much improved’ and ‘minimally improved’.

## Discussion

This report has demonstrated that mental health service, particularly clozapine treatment service, can be provided in a general hospital in a low-income setting. Although the number of patients who received the service was not large because of limited beds available for the service, the experience has resulted in important lessons that can easily be transferred into other similar hospitals which plan to start such services.

The majority of patients who received the service were men; this may be partly because of the larger number of male patients with schizophrenia in Ethiopia, which was demonstrated in the large epidemiological study.^4^ The other explanation could be that men have more severe course of schizophrenia needing treatment with clozapine.^7,26^ In studies from other countries, mainly high-income countries which extensively use clozapine for treating refractory cases of schizophrenia, patients who receive clozapine tend to be men.^27,28^

The majority of these patients were single and unemployed, a manifestation of severity of schizophrenia which causes significant disability.^7,29^ In high-income countries, these patients often rely on state welfare support, which amounts in billions of dollars; however, in low-income countries, such support is not available and patients rely on caregivers, causing significant burden on families.^30,31,32^ In this study, more than 82% of the patients were living with their families who provided everything the patients needed for their survival, including paying for their healthcare. The majority had to pay out of pocket for the healthcare their relatives received. Those patients who do not have families to look after them become vagrants, making them vulnerable to many adversities.^33^

Regarding indication for clozapine, the main indication was treatment-resistant schizophrenia, although some of the patients were put on clozapine for treatment of tardive dyskinesia, which resulted from treatment with first-generation antipsychotics. Interestingly, clozapine remains the single most important treatment for treatment-resistant schizophrenia, with seemingly no other alternative at the moment.^10,34^ Tardive dyskinesia was found to respond to clozapine in a number of studies. In this study, we did CGI score to track improvement, which was noted both in illness severity score and global impression of severity, the average global improvement score being below 2.68. The CGI score is used in many studies including in head-to-head trials comparing clozapine with first- and other second-generation antipsychotics for treatment-resistant schizophrenia, and response is usually defined as a mean global improvement score of 3 or less post-treatment.^35,36,37^ In patients who were admitted for treatment of tardive dyskinesia, serial AIMS score was done, and changes were observed between average AIMS scores at admission and discharge. The AIMS score is a useful tool for objectively assessing tardive dyskinesia, including severity of symptoms and impact.^24^ Significant reduction in the AIMS score was noted in our patients in the course of their treatment with clozapine. This finding is consistent with other studies that reported similar improvements, making clozapine an important intervention for a stigmatising and disabling side effect of antipsychotic drugs.^38,39^ The mechanism of action for this effect of clozapine was proposed to be because of hypersensitivity of dopaminergic receptors.^40^

Regarding side effects of clozapine, the majority of patients tolerated it well, with the most common side effects being sedation and constipation. Only three patients discontinued treatment because of cardiac side effects, all with persistent tachycardia and one with confirmed EEG abnormality, and the presence of cardiac side effects is an important reason for discontinuation of clozapine, which could suggest the presence of clozapine-induced myocarditis, reported to appear in as high as 3% of patients treated with clozapine.^41^ Patients suspected of myocarditis should discontinue treatment and be referred to specialist care with no option of rechallenge.^22^

Interestingly, ANC score remained within the normal range throughout the inpatient treatment; this could be explained by the relatively small number of participants, although the risk of agranulocytosis is highest in the first 3 months of treatment, but this cannot be a reason for complacency as it can happen anytime.^42,43^

### Challenges affecting clozapine service in Ethiopia

The clozapine treatment service was provided to patients living in the capital city. This renders the service inaccessible to the vast majority of patients who reside outside the capital. Even in the capital city, the service is available in only two hospitals.

The number of psychiatrists is less than 100, that is, less than 1 psychiatrist per million people, and more than 75% of the psychiatrists practise in the capital city.

Another challenge is affordability as most patients had to pay out of pocket for the service; this will significantly affect the vast majority of patients with treatment-resistant schizophrenia who are generally poor with no income and dependent on family members for their survival. Even while during treatment in the hospital, some of the services such as cardiac echocardiography and Electrocardiogram (EKG) as well as inflammatory markers were not readily available and unaffordable by many patients.

Although the presence of a national treatment guideline is a good step in the right direction, is there no mechanism for enforcement of the guideline. There is no system of registry of patients taking clozapine, nor there is a registry of professionals or pharmacies providing the service; this will make safety monitoring very challenging. Patients may get their ANC counts done in different laboratories, which will affect the quality of the result. There is no strict enforcement of ‘no blood, no drug’ in the pharmacies unlike the practice in high-income countries.

### Strengths and limitations of the study

This is the first report on the experience of setting up clozapine service in Ethiopia, and to the author’s knowledge amongst the few reports from low-income countries on this important intervention for treatment-resistant schizophrenia. It is expected to shed some light on the challenges and opportunities of initiating the service in a low-income setting, which will provide important benchmark for others to follow, especially in sub-Saharan Africa, where the service is nearly non-existent. The study limitations include small number of participants, which make the study underpowered, and the data presented come from retrospective chart review of the patients treated at the hospital. The CGI scale was rated retrospectively from progress reports on patient charts. The lack of perspectives on the service from different stakeholders is another limitation, which will be an important area of future research.

## Conclusions

Providing clozapine service was possible in a general hospital in Ethiopia where psychiatric service run by psychiatrists was present. Most patients with treatment-resistant schizophrenia and tardive dyskinesia showed significant improvement and, in general, side effects were infrequent, and the medication was well tolerated. The presence of a national treatment guideline was very useful, although mechanism to ensure implementation was not put in place. Accessibility and affordability will remain major challenges as most patients paid out of pocket, and the medication is available only in the capital city. Putting in place registry, making services freely available and scaling up of the service in referral hospitals outside the capital will potentially enable us to reach patients who desperately need this important treatment.
